# Development of the Referee Shared Mental Models Measure (RSMMM)

**DOI:** 10.3389/fpsyg.2020.550271

**Published:** 2020-10-19

**Authors:** Jorge Sinval, João Aragão e Pina, João Sinval, João Marôco, Catarina Marques Santos, Sjir Uitdewilligen, M. Travis Maynard, Ana Margarida Passos

**Affiliations:** ^1^Business Research Unit (BRU-IUL), Instituto Universitário de Lisboa (ISCTE-IUL), Lisbon, Portugal; ^2^William James Center for Research, ISPA – Instituto Universitário, Lisbon, Portugal; ^3^Faculdade de Medicina, Universidade de Lisboa, Lisbon, Portugal; ^4^Faculty of Philosophy, Sciences and Languages of Ribeirão Preto, University of São Paulo, São Paulo, Brazil; ^5^ISCTE Business School (IBS), Instituto Universitário de Lisboa (ISCTE-IUL), Lisbon, Portugal; ^6^Faculty of Economics, University of Porto, Porto, Portugal; ^7^Organisation, Strategy, and Entrepreneurship, Maastricht University School of Business and Economics, Maastricht University, Maastricht, Netherlands; ^8^Faculty of Psychology and Neuroscience, Maastricht University, Maastricht, Netherlands; ^9^Department of Management, College of Business, Colorado State University, Fort Collins, CO, United States

**Keywords:** shared mental models, referees, psychometrics, football, futsal

## Abstract

The concept of shared mental models refers to the shared understanding among team members about how they should behave in different situations. This article aimed to develop a new shared mental model measure, specifically designed for the refereeing context. A cross-sectional study was conducted with three samples: national and regional football referees (*n* = 133), national football referees and assistant referees and national futsal referees (*n* = 277), and national futsal referees (*n* = 60). The proposed version of the Referee Shared Mental Models Measure (RSMMM) has 13 items that are reflected on a single factor structure. The RSMMM presented good validity evidence both based on the internal structure and based on relations to other variables (presenting positive associations with team work engagement, team adaptive performance, and team effectiveness). Such promising psychometric properties point to an optimistic outlook regarding its use to measure shared mental models in futsal and football referee teams.

## Introduction

Shared mental models have been examined in numerous contexts (Resick et al., 2010a; Santos et al., 2015a, 2016; Tomás et al., 2017). However, one context where the role of shared mental models has received relatively little attention is sports referee teams (Filho and Tenenbaum, 2012; Aragão e Pina et al., 2018). This gap is interesting as football and futsal refereeing teams are highly interdependent in conducting their team tasks. Namely, they must coordinate several tasks before, during, and after the game (Samuel, 2015; Hancock et al., 2018); share technical and tactical knowledge to gain an adequate understanding of the task and match game needs (Mascarenhas et al., 2006; Mallo et al., 2012; McEwan and Beauchamp, 2014; Hancock et al., 2018); anticipate and adapt to the needs and actions of other members as well as changing task demands (Cannon-Bowers et al., 1993; Hancock et al., 2018); define a communication protocol to facilitate the team decision-making process (Cunningham et al., 2014; Samuel, 2015; Hancock et al., 2018) and engage in continuous learning together with the other team members (Collina, 2004; Cunningham et al., 2014; McEwan and Beauchamp, 2014). Nevertheless, to date, only one study has addressed the entire football refereeing team (see, Boyer et al., 2015), and currently there is no shared mental model scale adapted specifically for football and futsal refereeing teams.

Shared mental models refer to an organized and common understanding among team members regarding the essential aspects of work (Klimoski and Mohammed, 1994; Mohammed et al., 2010). Team members hold multiple mental models, about different domains, while they work on a task (Cannon-Bowers et al., 1993; Klimoski and Mohammed, 1994). Cannon-Bowers et al. (1993) proposed four types of models, namely, the equipment model, task model, team interaction model, and team model. The equipment model refers to knowledge about the equipment functioning, technology, and tools with which the team members interact. The task model refers to knowledge about the task procedures, task strategies, contingency plans, and environmental constraints. The team interaction model refers to knowledge about the roles and responsibilities of team members, role interdependencies, interaction patterns, and communication channels. The team model regards knowledge about task-relevant attributes of team members, such as knowledge, skills, abilities, preferences, and tendencies (Cannon-Bowers et al., 1993; Mathieu et al., 2000; Mohammed et al., 2010).

Nevertheless, Mathieu et al. (2000) merged those four models into two domains–task mental models (comprising the equipment and task models) and team mental models (comprising the team interaction and team models). Accordingly, over the years, researchers have most commonly analyzed task mental models and team mental models (Mathieu et al., 2000; Lim and Klein, 2006; Santos and Passos, 2013). Task mental models refer to a similar understanding among team members about work objectives, team resources, task procedures and practices, and task duties. Team mental models refer to a similar understanding among team members about interpersonal interaction, team members’ roles and responsibilities, and role interdependencies (Mathieu et al., 2000; Mohammed et al., 2010). In this article, this distinction between task and team mental models was made, as it has received extensive support from empirical studies (Mathieu et al., 2000; Lim and Klein, 2006; Santos and Passos, 2013; Santos et al., 2015a). Another dimension recently proposed by Randall et al. (2011) was also considered, namely, strategy mental models, which refer to “an understanding of strategic priorities, the trade-offs, and relationships among strategic alternatives, and the implications of strategic decisions” (p. 527).

Drawing on research on shared mental models in organizational teams, it is arguable that referee teams that develop shared mental models can anticipate each other’s needs and adapt their behaviors to fit tightly to task demands (Cannon-Bowers et al., 1993; Mohammed et al., 2010). When the referee team members develop a common understanding on the tools and technologies they interact with, such as audio communication system, electronic flags, or video assistant referees (VARs), on the task procedures and strategies, as well as on the strategic priorities, they similarly, interpret the cues and make effective and quick decisions on the field (Kellermanns et al., 2008; Randall et al., 2011). Furthermore, when referee team members develop a shared understanding regarding each other’s roles and responsibilities, as well as on the knowledge, skills, and abilities of each other, this enables them to effectively communicate and work in a coordinated way, which allows them to adapt to unexpected events, and perform their tasks successfully (Mathieu et al., 2000; Muponde and Muchemwa, 2011; Santos and Passos, 2013; Boyer et al., 2015; Diotaiuti et al., 2017; Uitdewilligen et al., 2018). Some psychometric instruments to measure mental models have already been proposed: the Shared Mental Model Scale (SMMS; Santos et al., 2015a) also with a shorter unidimensional version (Santos et al., 2015b), the Team-Related Knowledge Measurement Instrument (TeamKMI; Johnson et al., 2007), and the Perceived Mutual Understanding (PMU) scale (Burtscher and Oostlander, 2019). Yet, none of the existing instruments has been tested among referees. Therefore, by developing a shared mental model measure for referees, scholars could begin to examine shared mental models within the context of referee teams and allow further examination of their antecedents and outcomes (Aragão e Pina et al., 2021).

### Research Hypotheses

One of the most desirable psychometric properties of an instrument is its dimensionality stability across different samples (Nunnally and Bernstein, 1994). As so, if an instrument maintains its dimensionality with a good fit to the different sample datasets, one can assume that the items and factors proposed are adequate to measure the desired construct/s over different groups of individuals. It is particularly important to have dimensionality evidence when independent samples of the same population are analyzed with the same instrument (Marôco, 2014). Because the perceptions of mental models can vary from context to context, it is expected that the dimensionality of the proposed measure may have a different number of factors in comparison with the instrument in which this measure was initially based (i.e., three factors). However, it is assumed that the proposed dimensionality will present a good fit to the data (H1; i.e., three different samples). It is expected that the proposed dimensionality on the first sample data will be reproduced with a good fit in the two other independent samples. If such dimensionality (i.e., factor structure) holds in all the samples, there will be promising evidence of dimensionality (American Educational Research Association, American Psychological Association, and National Council on Measurement in Education, 2014).

Another important psychometric property is the reliability evidence, which can be assessed through internal consistency (Nunnally and Bernstein, 1994). Previous instruments measuring mental models reported acceptable values of internal consistency, as the PMU (Burtscher and Oostlander, 2019) with α = 0.83 and ω = 0.83. The TeamKMI reported globally satisfactory internal consistency estimate values (Johnson et al., 2007). Moreover, the SMMS reported satisfactory internal consistency values (Santos et al., 2015b). The second hypothesis (H2) presumes the Referee Shared Mental Models Measure (RSMMM) shows good evidence of the scores’ reliability, more specifically in terms of internal consistency (Nunnally and Bernstein, 1994). Such estimates should be desirably high (i.e., ≥ 0.70; Iacobucci and Duhachek, 2003). Adequate internal consistency values will indicate that the items are measuring the same construct, measuring the construct consistently (McDonald, 1999).

The third hypothesis (H3) assumes that the RSMMM will present measurement invariance among referees from different sports. Such property is essential to directly compare groups within the same instrument (Davidov et al., 2014). Measurement invariance has been tested before among referees of different types of sports in a measure of self-efficacy (Myers et al., 2012), also among referees and assistant referees in football (Brandão et al., 2014) and also between elite and non-elite football referees (Johansen et al., 2018).

The extent of the relations of an instrument’s scores with external variables constitutes a critical source of validity. This particular source of validity is denominated as validity evidence based on the relation to other variables (American Educational Research Association, American Psychological Association, and National Council on Measurement in Education, 2014). As such, some related constructs are expected to be associated with shared mental models. Team work engagement is an affective–motivational construct that is expected to be positively related to shared mental models because higher team work engagement means higher team enthusiasm and energy (Costa et al., 2014b). The mental models construct is a cognitive one, which is expected to enhance team members’ anticipation of actions and communication, conducting to positive feelings. This is also true regarding team effectiveness, because a higher common understanding of the way the team works will allow predicting behavior patterns that will likely increase the effectiveness of the team (Marks et al., 2002; Mathieu et al., 2009; DeChurch and Mesmer-Magnus, 2010). As such, a positive association between mental models and team effectiveness is expected to be observed. Associated to a higher level of shared mental models is expected to be a higher perception of team adaptive performance. Team members with shared cognitive representations regarding team function will predict the other team members move straightforwardly and consequently improving the ability to react and adjust when necessary (Pulakos et al., 2006). As so, the fourth hypothesis (H4) establishes that the suggested shared mental models measure will present validity evidence based on the relation to other variables, namely, nomological evidence in convergent terms with team work engagement, team effectiveness, and team adaptive performance.

## Materials and Methods

### Sample

This article uses data from three different studies with non-probabilistic convenience samples where data were collected within a cross-sectional survey at the individual level; all participants are Portuguese football referees or assistant referees or futsal referees. Depending on the tournament, football referee teams range from three to seven members, whereas futsal referee teams range from three to four members. Team members within each team are usually the same, with some exceptions (i.e., injuries, not being considered apt in physical or written examinations). However, in the case of the top-class football national referees (i.e., C1 class), rotation between team members is more frequent.

#### Study I

The sample I data (*n* = 133) were constituted by national football referees (*n* = 67), with mean_age_ = 30.02 (*SD*_age_ = 3.16) years, mean_experience_ = 12.29 (*SD*_experience_ = 3.15) years; and football regional referees (*n* = 66) mean_age_ = 26.37 (*SD*_age_ = 3.21) years, mean_experience_ = 8.89 (*SD*_experience_ = 3.15). All referees completed the questionnaires.

#### Study II

The sample II data (*n* = 277) were composed by football national referees (*n* = 135) with mean_experience_ = 12.44 (*SD*_experience_ = 5.06) years, futsal national referees (*n* = 117) with mean_experience_ = 11.71 (*SD*_experience_ = 4.88) years, and football national assistant referees (*n* = 25) with mean_experience_ = 18.44 (*SD*_experience_ = 4.16) years. All referees completed the questionnaires.

#### Study III

The sample III data (*n* = 60) had only futsal national referees with mean_age_ = 34.54 (*SD*_age_ = 5.52) years, mean_experience in the current team_ = 2.72 (*SD*_experience in the current team_ = 3.13) years.

### Measures

All the self-report measures were collected at the individual level, reflecting the perceptions of the subject about the team.

#### Shared Mental Models

Shared mental models refer to a multidimensional construct. In this article, three dimensions were considered, namely, task mental models, team mental models, and strategy mental models. Referees must develop a similar understanding of the task procedures, practices, and strategies to make decisions, likely scenarios and contingencies (Cannon-Bowers et al., 1993; Mathieu et al., 2000; Aragão e Pina et al., 2019), and contingency plans (Mohammed et al., 2010). Regarding the task mental models, referee team members must develop a similar understanding about the equipment functioning and equipment limitations (Cannon-Bowers et al., 1993), as well as about the technology and tools with which they interact to make decisions (Mathieu et al., 2000; Mohammed et al., 2010). Example of such equipment are the audio communication system, the Video Assistant Referee (VAR), or the goal-line technology (GLT). Referee team members must also develop a similar understanding of the environmental constraints and the aspects of the task environment that affect team performance (Cannon-Bowers et al., 1993).

Concerning the team mental models, referee team members must develop a similar understanding about the roles and responsibilities of each team member, the role interdependencies, and about interaction patterns and communication channels and patterns (Cannon-Bowers et al., 1993; Mathieu et al., 2000; Mohammed et al., 2010). Besides, they must develop a similar understanding about the knowledge, skills, and abilities of each team member and about the team members’ preferences to make decisions during the games (Cannon-Bowers et al., 1993; Mathieu et al., 2000; Aragão e Pina et al., 2019). Regarding the strategy mental models, referee team members must develop a similar understanding of the strategic priorities, as well as the implications of strategic decisions (Randall et al., 2011).

This measure was named as RSMMM (Table 1). Based on relevant literature on shared mental models, namely, in other instruments (Santos et al., 2015a,b), an initial pool of 13 items was developed across the three dimensions: task (e.g., “In my team, the team members have a similar understanding about the technology and tools needed to make decisions during a game”); team (e.g., “In my team, the team members have a similar understanding about the knowledge, skills, and abilities of each other”); and strategy (e.g., “In my team, the team members have a similar understanding about the strategic priorities of the game”). It was ensured that the shared mental models’ items, in particular, the items of the task dimension, were specific to the context of referee teams (Cannon-Bowers and Salas, 2001) by stating that team members have the knowledge needed to make decisions during a game or by providing examples related to the referees’ responsibilities. For instance, “In my team, the team members have a similar understanding about resources needed to make decisions during a game” and “In my team, the team members have a similar understanding about the tasks each team member has to do (e.g., train during the week, prepare the game properly, employ an exemplary behavior, make a difficult decision).” Each item was scored on a seven-point Likert scale (1 = “Totally disagree”, 2 = “Strongly disagree”, 3 = “Disagree”, 4 = “Neither agree, nor disagree”, 5 = “Agree”, 6 = “Strongly agree”, 7 = “Totally agree”).

**TABLE 1:** Referee Shared Mental Models Measure (RSMMM) items.

Item	English version of RSMMM							Portuguese (Portugal) version of RSMMM						
	Totally disagree	Strongly disagree	Disagree	Neither disagree nor agree	Agree	Strongly agree	Totally agree	Discordo totalmente	Discordo muito	Discordo em parte	Nem concordo, nem discordo	Concordo em parte	Concordo muito	Concordo totalmente
	**1**	**2**	**3**	**4**	**5**	**6**	**7**	**1**	**2**	**3**	**4**	**5**	**6**	**7**

	**Shared Mental Models**							**Modelos Mentais Partilhados**						

1	In my team, members have a similar understanding of the resources that are needed to make decisions during a game.							Na minha equipa, os membros têm um entendimento semelhante sobre os recursos que são necessários para tomar as decisões durante um jogo.						
2	In my team, members have a similar understanding of the technology and tools needed to make decisions during a game.							Na minha equipa, os membros têm um entendimento semelhante sobre a tecnologia e as ferramentas necessárias para tomar as decisões durante um jogo.						
3	In my team, members have a similar understanding of the procedures and practices needed to make decisions during a game.							Na minha equipa, os membros têm um entendimento semelhante sobre os procedimentos e práticas necessários para tomar as decisões durante um jogo.						
4	In my team, even when we are confronted with incidents or problems related to our performance, we have a similar understanding of how to perform our tasks.							Na minha equipa, mesmo quando somos confrontados com incidentes ou problemas relacionados com a nossa atuação, temos um entendimento semelhante sobre como realizar as nossas tarefas.						
5	In my team, members have a similar understanding of what they must do (e.g., train during the week, properly prepare the game, adopt exemplary behavior, make a difficult decision).							Na minha equipa, os membros têm um entendimento semelhante em relação ao que cada um tem que fazer (ex.: treinar durante a semana, preparar adequadamente o jogo, adotar um comportamento exemplar, tomar uma decisão difícil).						
6	In my team, members have a similar understanding of how their roles are related.							Na minha equipa, os membros têm um entendimento semelhante sobre a forma como os papéis de cada um estão relacionados.						
7	In my team, members have a similar understanding of how to interact with each other.							Na minha equipa, os membros têm um entendimento semelhante sobre a forma como interagir uns com os outros.						
8	In my team, members have a similar understanding of what the best methods are for communicating with each other.							Na minha equipa, os membros têm um entendimento semelhante sobre quais os melhores métodos para comunicar uns com os outros.						
9	In my team, members have a similar understanding of each other’s knowledge, skills and abilities.							Na minha equipa, os membros têm um entendimento semelhante em relação aos conhecimentos, competências e capacidades de cada um.						
10	In my team, members have a similar understanding of each other’s preferences, which are relevant to making decisions during a game.							Na minha equipa, os membros têm um entendimento semelhante em relação às preferências de cada um, que são relevantes para tomar as decisões durante um jogo.						
11	In my team, members have a similar understanding of the game’s strategic priorities.							Na minha equipa, os membros têm um entendimento semelhante em relação às prioridades estratégicas do jogo.						
12	In my team, members have a similar understanding of the implications of the strategic decisions that are made.							Na minha equipa, os membros têm um entendimento semelhante em relação às implicações das decisões estratégicas que são tomadas.						
13	In my team, members have a similar understanding of which aspects of the game are most important to team performance.							Na minha equipa, os membros têm um entendimento semelhante sobre quais os aspetos do jogo que são mais importantes para o desempenho da equipa.						

#### Team Work Engagement

Team work engagement is defined as an emergent state that develops from team members’ interactions and that cannot be found in individuals being exclusive to teams (Costa et al., 2016). To measure team work engagement, the Team Work Engagement Scale was used (Costa et al., 2014a). This instrument consists of nine items measured in a seven-point Likert scale (1 = “Totally disagree”, 7 = “Totally agree”). Team Work Engagement is seen as a second-order factor (as the individual measure; Sinval et al., 2018b, a) that comprises three first-order dimensions (i.e., vigor, dedication, and absorption). This instrument showed good validity evidence based on the internal structure in previous studies, namely, in terms of reliability, having Cronbach’s α of 0.85 a 0.97 for the vigor factor, 0.88 and 0.95 for the dedication factor; and 0.83 and 0.95 for the absorption factor (Costa et al., 2014a). Examples of items are as follows: “At our work, we feel bursting with energy” (vigor), “We are enthusiastic about our job” (dedication), and “We feel happy when we are working intensely” (absorption).

#### Team Adaptive Performance

Team adaptive performance is defined as an emergent state that occurs as a consequence of the adaptation process, in which individuals and teams cope with the demands of the context (Maynard et al., 2015). The Team Adaptive Performance Scale was used to measure team adaptive performance (Marques-Quinteiro et al., 2015). This instrument has eight items that were answered using a seven-point Likert scale (1 = “Totally disagree”, 7 = “Totally agree”). This instrument assumes that team adaptive performance is a second-order latent variable with two first-order latent factors (factor I: problem-solving–oriented factor, six items; and factor II: learning work tasks, technologies, and procedures factor, two items). Examples of items are as follows: “We use creative ideas to manage incoming events” (problem-solving–oriented), and “We remain calm and behave positively under highly stressful events” (learning work tasks, technologies, and procedures).

#### Team Effectiveness

Team effectiveness is conceived in three criteria: team performance, quality of group experience, and team viability (Aubé and Rousseau, 2005). Team performance has been seen in the function of the assigned team goals (Hackman, 1987). The quality of group experience is defined as the positiveness of the social climate in the team (McGrath, 1991). The team viability consists in the capacity of the team to adapt to external and internal changes and also to the likelihood of team members continuing to work together (Hackman, 1987). The team effectiveness dimension was measured using the Portuguese version of the Scale of Effectiveness of Teams (3Es; Vicente et al., 2014). This instrument has three first-order factors (team performance, quality of group experience, and team viability), which are explained by a hierarchical structure (second-order factor) called effectiveness. The items were scored with a Likert scale from 1 = “Totally disagree” to 7 = “Totally agree”. In the original version with the Canadian sample (Aubé and Rousseau, 2005), the authors studied the internal consistency, and good Cronbach’s α values were evidenced (α_team performance_ = 0.82, α_team viability_ = 0.84, α_quality of group experience_ = 0.96). Examples of items are as follows: “The members of this team attain their assigned performance goals” (team performance); “The social climate in our work team is good” (quality of group experience); and “Team members adjust to the changes that happen in their work environment” (team viability).

### Procedure

For samples I and II studies, the institutional review board, and the National Referees’ Committee approved the study. National referees were attending a seminar, and regional referees were attending a promotion seminar compulsory for those wishing to be considered for promotion to the national level. Data were collected at the beginning of each seminar, after providing a brief explanation of the nature of the investigation. The institutional approval of the Portuguese Football Federation was obtained for sample III’s study. All referees participated voluntarily, and written or electronic informed consent was obtained from all participants, and confidentiality for their responses was ensured.

### Data Analysis

All statistical analyses were performed with *R* (R Core Team, 2020) through RStudio (RStudio Team, 2020). The descriptive statistics were obtained with the *skimr* package (McNamara et al., 2018); the coefficient of variation (*CV*) was calculated through the *sjstats* package (Lüdecke, 2019), and the standard error of the mean (*SEM*) was estimated by the *plotrix* package (Lemon, 2006). The mode was calculated with the *DescTools* package (Signorell et al., 2019). Severe univariate normality violations were considered for absolute values of *sk* > 3 and *ku* > 7 (Finney and DiStefano, 2013; Marôco, 2014).

Regarding the exploratory factor analysis (EFA), the Kaiser–Meyer–Olkin (*KMO*) coefficient was used as a measure of sampling adequacy (Kaiser and Rice, 1974). The Bartlett test (Bartlett, 1951) was chosen to test if the correlation matrix was factorable (i.e., the correlations differ from 0) (Revelle, 2019). *KMO* values > 0.8 and Bartlett test significance ≤ 0.05, indicating adequate sampling (Marôco, 2018). The number of factors was determined through the comparison data (CD) approach, as suggested by Ruscio and Roche (2012), which stated that this technique outperforms Parallel Analysis. CD is a variant of Parallel Analysis that reproduces the correlation matrix rather than generating random data (Courtney, 2012). The extraction of the factors was performed using the principal components analysis with a weighted least-squares factoring method on the *polychoric* correlation (ρ*_PC_*) matrix with oblimin rotation and weighted least-squares factoring. The cutoff for items’ loadings was 0.40. The CD analysis was conducted using the *RGenData* package (Ruscio, 2018). The Bartlett test, the KMO coefficient, factors’ extraction and the ρ*_PC_* were produced using the *psych* package (Revelle, 2019). As goodness-of-fit index for the EFA, the RMSR (root mean square of the residual) was used.

Confirmatory factor analysis (CFA) was conducted with the *lavaan* package (Rosseel, 2012) using the weighted least-squares means and variances (WLSMV) estimation method for ordinal variables (Muthén, 1983). As goodness-of-fit indices, the *TLI* (Tucker–Lewis index), *NFI* (normed fit index), χ^2^/df (ratio chi-square and degrees of freedom), *CFI* (comparative fit index), the *RMSEA*, and the *SRMR* (standardized root mean square residual) were used. For values of χ^2^/df < 5, values of *CFI, NFI*, and *TLI* > 0.95; values of *SRMR* < 0.08; and *RMSEA* < 0.08, the fit of the model was considered good (Hoyle, 1995; Boomsma, 2000; McDonald and Ho, 2002; Byrne, 2010; Marôco, 2014).

To analyze the convergent validity evidence, the average variance extracted (*AVE*) was estimated (Fornell and Larcker, 1981). For values of *AVE* ≥ 0.5 (Hair et al., 2019), adequate convergent validity evidence was assumed.

The discriminant validity evidence was tested to verify whether the items that represent a dimension were strongly correlated with other dimensions. To assess such evidence, the Fornell and Larcker’s (1981) approach was used: for two factors, *x* and *y*, if *AVE_x_* and *AVE_y_* ≥ ρ^2^*_xy_* (squared correlation between the factors *x* and *y*), adequate discriminant validity evidence is assumed.

The reliability of the scores was assessed with estimates of internal consistency, α (Cronbach, 1951), and ω (Raykov, 2001), using the *semTools* package (Jorgensen et al., 2019), where higher values were indicative of better internal consistency results. The α coefficient was calculated using the polychoric correlation matrix. The second-order reliability estimates were as follows: the proportion of the second-order factor explaining the total score (ω_L1_), the proportion of variance explained by second-order factor after partialing the uniqueness of the first-order factor (ω_partialL1_), and the variance of the first-order factors explained by the second-order factor (ω_L2_). Such reliability estimates were obtained with the *semTools* package (Jorgensen et al., 2019). The confidence intervals (CIs) for the internal consistency estimates were obtained through the *userfriendlyscience* package (Peters, 2018) and the *boot* package (Davison and Hinkley, 1997; Canty and Ripley, 2020) using 1,000 bootstrap replicates. The bias-corrected and accelerated method was used, which tend to provide better coverage in non-normal sampling distributions (Efron and Tibshirani, 1994; Carpenter and Bithell, 2000).

The measurement invariance was assessed and verified using the *lavaan* package (Rosseel, 2012) and the *semTools* package (Jorgensen et al., 2019). A group of five models was compared: (a) configural invariance; (b) first-order factor loadings; (c) thresholds/intercepts of measured variables (depending on if the items are considered or not as categorical); (d) residual variances of observed variables; and (e) latent means. The latent variable means were compared, and Cohen *d* was used as the effect size (Cohen, 1988).

## Results

The presented results refer to three different studies with three different samples. First, the three samples were merged, and the instrument’s expected dimensionality analyzed. Subsequently, the samples were individually analyzed to obtain different validity evidence from each of them.

### Merge Samples

#### Validity Evidence Based on the Internal Structure

The dimensionality, reliability of scores, and measurement invariance of the instrument will be tested to verify the robustness of this source of validity evidence.

##### Items’ distributional properties

As Table 2 shows, none of the items for samples I and II presented severe problems of univariate normality because all of them presented |*sk*| < 3 and |*ku*| < 7 (Finney and DiStefano, 2013; Marôco, 2014). However, some of sample III items’ absolute values of *ku* were greater than 7 (i.e., items 1, 2, 3, 6, and 9; Table 2). Item 5 was the one that presented more variability (i.e., *CV*) in the answers in all samples.

**TABLE 2:** Items’ distributional properties.

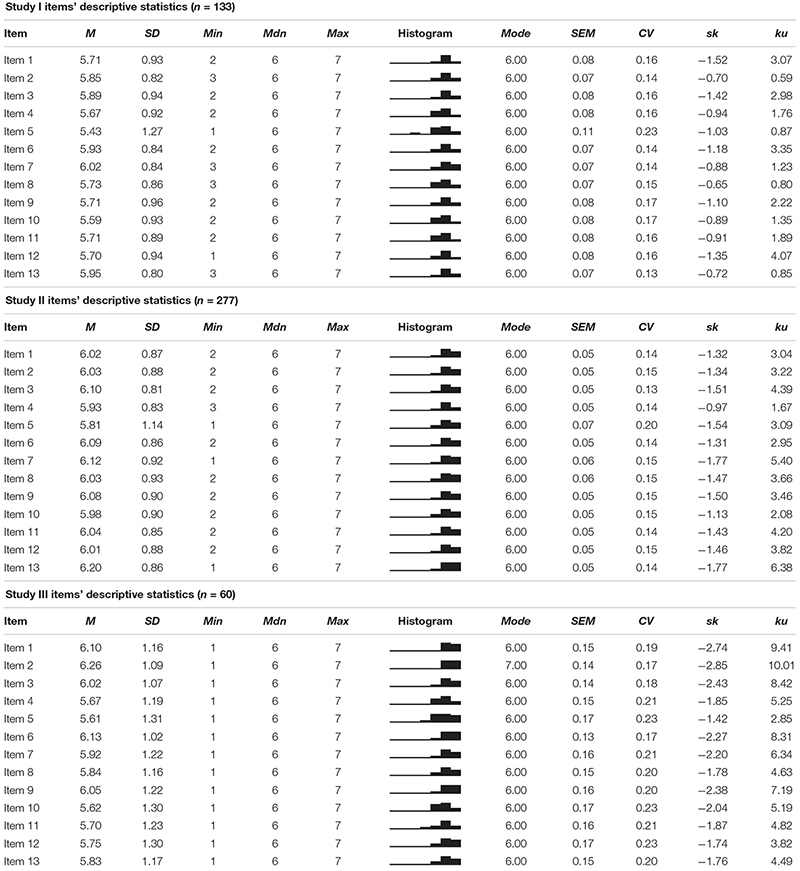

Following the recommendations of Finney et al. (2016) with categorical items with six or more points, both maximum likelihood estimation with robust (Huber–White) standard errors (MLR) and diagonal weighted least-squares methods (as the WLSM estimator) can be used. The WLSMV estimator was chosen because it does not require multivariate normality as an assumption. To analyze the validity evidence based on the internal structure of the new measure, several steps were carried (i.e., dimensionality, reliability, and measurement invariance).

##### Dimensionality

To test the expected three first-order factors of the RSMMM, a CFA was conducted with all the available data from the three collected samples. The CFA is the most appropriate technique to use when there is a definite theory regarding the latent factors and their relationships to the indicators, that is, dimensionality (Brown, 2015; Finch and French, 2015). Items 1 to 4 were used as indicators of the task factor; items 5 to 10 were expected to be indicators of the dimension team, and items 11 to 13 were developed as potential indicators of the latent variable strategy.

The goodness-of-fit indices were indicative of good fit to the data (χ^2^(62) = 184.686, *n* = 526, χ^2^/df = 2.979, *CFI* = 0.999, *NFI* = 0.998, *TLI* = 0.998, *SRMR* = 0.031, *RMSEA* = 0.061, *P*(rmsea ≤ 0.05) = 0.032, 90% CI ]0.051; 0.072[). The convergent validity evidence based on the internal structure was good (*AVE*_task_ = 0.78, *AVE*_team_ = 0.70, *AVE*_strategy_ = 0.85). However, the discriminant validity evidence based on the internal structure was not satisfactory, because the latent correlations between the factors were too high (*r*_task × team_ = 0.919, *p* < 0.001; *r*_task × strategy_ = 0.870, *p* < 0.001; *r*_team × strategy_ = 0.915, *p* < 0.001). Comparing the values of the *AVE* of each pair of factors with their squared correlation value, only one of the three pairs (task and strategy) showed evidence of discriminant validity. The *r*^2^_task × team_ = 0.845 was greater than *AVE*_task_ = 0.78 and *AVE*_team_ = 0.70; the *r*^2^_task × strategy_ = 0.757 was smaller than *AVE*_task_ = 0.78 and *AVE*_strategy_ = 0.85; and *r*^2^_team × strategy_ = 0.838 was greater than *AVE*_team_ = 0.70, but smaller than *AVE*_strategy_ = 0.85. Such finding might be indicative of a unidimensional model, which should be investigated through the appropriate analysis (i.e., EFA).

##### Reliability of the scores: Internal consistency

The merged data of the three different studies revealed good reliability evidence in terms of internal consistency (α_task_ = 0.93, 95% CI ]0.91; 0.94[; ω_task_ = 0.87, 95% CI ]0.82; 0.90[; α_team_ = 0.93, 95% CI ]0.91; 0.94[; ω_team_ = 0.90, 95% CI ]0.88; 0.91[; α_strategy_ = 0.94, 95% CI ]0.92; 0.95[; ω_strategy_ = 0.90, 95% CI ]0.88; 0.92[).

Because the content explained by the three different factors is similar, the dimensionality was investigated using an exploratory approach (EFA), where the EFA’s suggested dimensionality from sample I was then tested (through CFA) in samples II and III’s data.

When the empirical evidence lacks regarding the construct expected dimensionality, EFA might be most appropriate than CFA (Finch and French, 2015). The EFA attributes a small burden on the researcher concerning the latent factors and their relationships to the indicators, making possible establishing an interval of the number of factors that can emerge from the indicators (Marôco, 2018).

### Study I

#### Validity Evidence Based on the Internal Structure

##### Dimensionality

**FIGURE 1:**
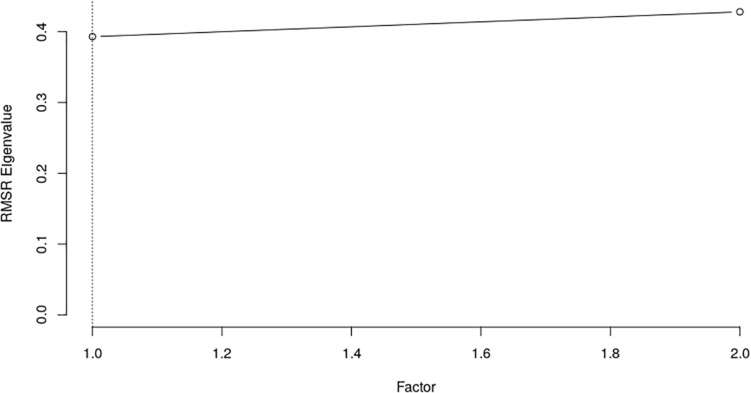
Fit to comparison data (*n* = 133). The CD analysis suggested that the number of factors to retain is one.

Data obtained from study I met the *KMO* coefficient (0.900) and Bartlett test of sphericity (χ^2^(78) = 963.521; *p* < 0.001). The CD suggested that the best solution contains only one factor (Figure 1).

The one-factor solution was adopted, and the results of the correspondent EFA (Table 3) revealed 50.8% of explained variance (*RMSR* = 0.086).

**TABLE 3:** Exploratory factor analysis loadings and total of explained variance.

Items	Factor 1
Item 1	0.826
Item 2	0.779
Item 3	0.762
Item 4	0.631
Item 5	0.440
Item 6	0.697
Item 7	0.731
Item 8	0.805
Item 9	0.677
Item 10	0.685
Item 11	0.721
Item 12	0.724
Item 13	0.713
Total of variance	0.508

##### Reliability of the scores: Internal consistency

The study I’s data revealed good reliability evidence in terms of internal consistency (α = 0.93, 95% CI ]0.91; 0.95[; ω = 0.93, 95% CI ]0.91; 0.95[).

To test the proposed structure observed in study I’s sample and see if it was adequate for a second and third independent samples from the population, a CFA was also performed on study II and study III samples.

### Sample II

As previously mentioned, the use of CFA demands strong theoretical and/or empirical evidence regarding the dimensionality of a psychometric instrument. As such, because study I’s sample provided empirical evidence supporting the one-factor solution, the CFA will be used to investigate the RSMMM single-factor model (Finch and French, 2015).

#### Validity Evidence Based on the Internal Structure

##### Dimensionality

**FIGURE 2:**
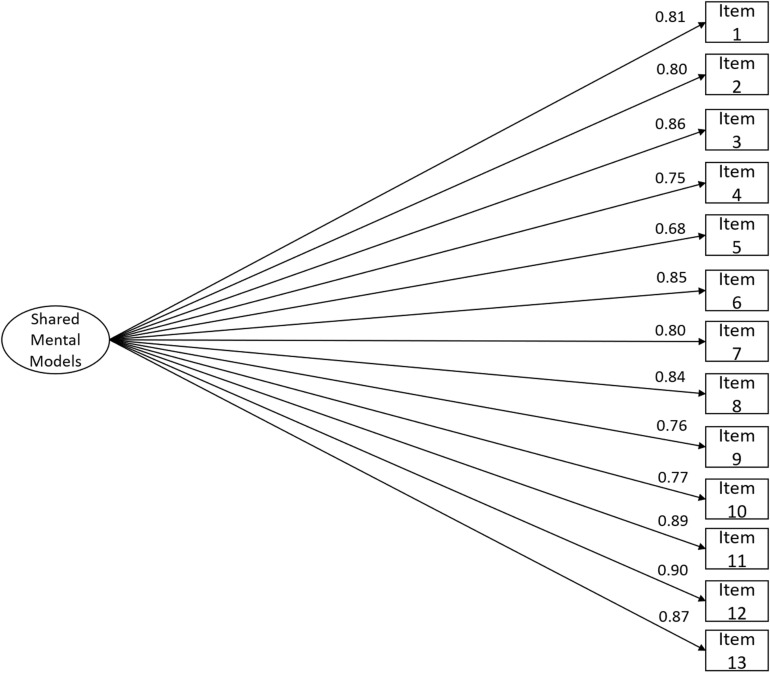
RSMMM one-factor version (13-item) structure fit using study II’s sample (*n* = 277). Factor loadings for each item are shown: χ^2^(65) = 271.199, *n* = 277, χ^2^/df = 4.172, *CFI* = 0.993, *NFI* = 0.991, *TLI* = 0.992, *SRMR* = 0.054, *RMSEA* = 0.107, *P*(rmsea ≤ 0.05) < 0.001, 90% CI ]0.094; 0.121[.

The goodness-of-fit indices were indicative of an acceptable fit of study II’s data to the model (Figure 2; χ^2^(65) = 271.199, *n* = 277, χ^2^/df = 4.172, *CFI* = 0.993, *NFI* = 0.991, *TLI* = 0.992, *SRMR* = 0.054, *RMSEA* = 0.107, *P*(rmsea ≤ 0.05) < 0.001, 90% CI ]0.094; 0.121[). In terms of convergent validity based on the internal structure, the estimate of *AVE* was good (*AVE* = 0.67).

##### Reliability of the scores: Internal consistency

Regarding study II’s internal consistency, the obtained values revealed good validity evidence in terms of reliability (α = 0.96, 95% CI ]0.95; 0.97[; ω = 0.92, 95% CI ]0.89; 0.93[). Both the α and ω coefficients were indicative of good evidence in terms of the reliability of the scores.

##### Measurement invariance

Measurement invariance between sports refereed (i.e., football and futsal) was tested using study II’s sample. Because there were only 25 football assistant referees, the measurement invariance analysis was performed only with the futsal and football referees. To conduct the measurement invariance considering the ordinal nature of the items, it is required that the items in both groups have the same number of thresholds. Because both groups had a different number of thresholds for some items, it was not possible to use WLSMV. As so, the measurement invariance analysis was performed using the MLR estimator because this method has been shown to work well with categorical data with no severe deviations from the normal distribution (Rhemtulla et al., 2012). As Table 4 shows, full uniqueness measurement invariance was achieved both by the Δ*CFI* and Δχ^2^ criteria (Satorra and Bentler, 2001; Cheung and Rensvold, 2002), which allows establishing comparisons between the shared mental models latent scores among the football and futsal referees.

**TABLE 4:** Measurement invariance analysis among futsal and football referees (study II’s sample).

Model	χ^2^	*df*	χ^2^/df	*CFI*_*robust*_	Δχ^2^	Δ*CFI*_*robust*_
**Sport refereed**						
Configural	436.77	130	3.36	0.888	–	–
Metric	442.68	142	3.12	0.893	4.071^*ns*^	0.005
Scalar	455.31	154	2.96	0.892	13.485^*ns*^	−0.001
Full uniqueness	495.30	167	2.97	0.888	16.641^*ns*^	−0.004
Latent means	499.45	168	2.97	0.887	5.495*	−0.002

^*ns*^*p* < 0.05; *p ≤ 0.05.

The shared mental models’ latent means presented significant differences among the futsal and football referees (Δχ^2^(1) = 5.495, *p* = 0.019, *d* = 0.168), with the football referees perceiving higher levels of shared mental models than their futsal counterparts.

### Sample III

#### Validity Evidence Based on the Internal Structure

##### Dimensionality

**FIGURE 3:**
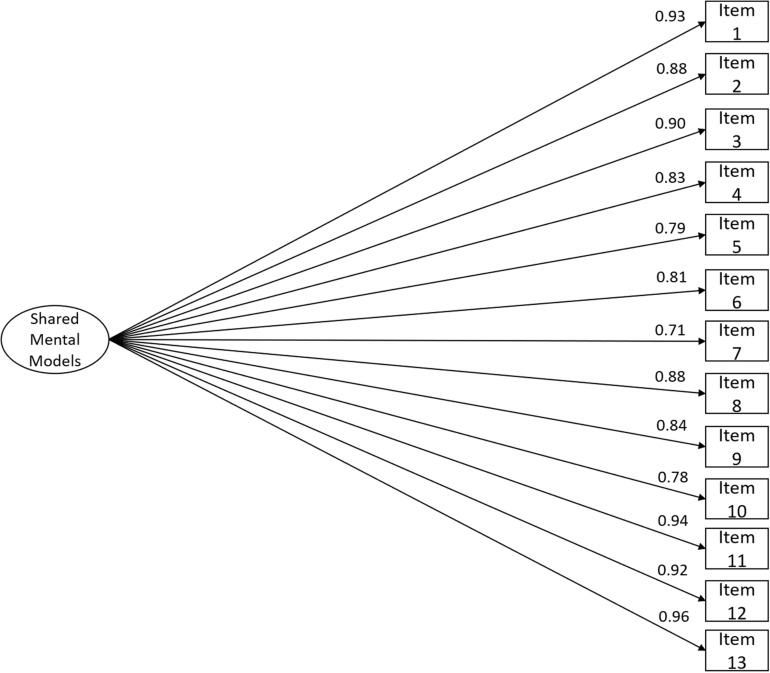
RSMMM one-factor version (13-item) structure fit using study III’s sample (*n* = 60). Factor loadings for each item are shown: χ^2^(65) = 74.428, *n* = 60, χ^2^/df = 1.145, *CFI* = 0.999, *NFI* = 0.994, *TLI* = 0.999, *SRMR* = 0.059, *RMSEA* = 0.050, *P*(rmsea ≤ 0.05) = 0.482, 90% CI ]0.000; 0.095[.

Study III’s CFA revealed an excellent fit to the data (Figure 3; χ^2^(65) = 74.428, *n* = 60, χ^2^/df = 1.145, *CFI* = 0.999, *NFI* = 0.994, *TLI* = 0.999, *SRMR* = 0.059, *RMSEA* = 0.050, *P*(rmsea ≤ 0.05) = 0.482, 90% CI ]0.000; 0.095[). The convergent validity evidence in terms of the internal structure was particularly good (*AVE* = 0.74).

##### Reliability of the scores: Internal consistency

The internal consistency estimates of study III’s sample were like study I’s and study II’s ones (α = 0.98, 95% CI ]0.93; 0.99[; ω = 0.98, 95% CI ]0.93; 0.99[) and as so were indicative of good evidence in terms of the reliability of the scores.

#### Validity Evidence Based on the Relation With Other Variables

The validity evidence based on the relation to other variables was investigated using study III’s sample. The nomological evidence was verified (i.e., convergent validity with team adaptive performance, team work engagement, and team effectiveness). For such analysis, the structural equation model framework was used.

##### Measurement model

Because the sample size (i.e., *N*_study III_ = 60) was too small to be used with the WLSMV estimator on this model, the MLR estimator was used in all subsequent analyses. The measurement model of the team work engagement measure revealed an acceptable fit to the data (χ^2^(25) = 69.366, *n* = 54, χ^2^/df = 2.775, *CFI* = 0.932, *NFI* = 0.900, *TLI* = 0.902, *SRMR* = 0.045, *RMSEA* = 0.181, *P*(rmsea ≤ 0.05) < 0.001, 90% CI ]0.131; 0.233[) after constraining the error variance of the first-order factor (dedication) to 0.01 in order to avoid negative variance. The second-order reliability estimates were good (ω_L1_ = 0.95, 95% CI ]0.87; 0.99[; ω_partialL1_ = 0.97, 95% CI ]0.92; 0.99[; ω_L2_ = 0.98, 95% CI ]0.93; 1.00[).

The hierarchical model of the team adaptive performance scale revealed an acceptable fit to the data (χ^2^(19) = 44.166, *p* = 0.001, *n* = 54; χ^2^/df = 2.325; *CFI* = 0.957; *TLI* = 0.936; *NFI* = 0.928; *SRMR* = 0.034; *RMSEA* = 0.157; *P*(rmsea ≤ 0.05) = 0.004, 90% CI ]0.096; 0.217[) after adding one correlation path between item 5’s and item 6’s residuals (*r* = 0.636; *p* = 0.024). The variance of the problem-solving–oriented factor was constrained to 0.01 to avoid negative variances. The structural weights (γ) of the two factors were constrained to be equal, to solve the model identification problem of two first-order factors in a hierarchical model. The second-order reliability estimates were good (ω_L1_ = 0.96, 95% CI ]0.87; 0.98[; ω_partialL1_ = 0.97, 95% CI ]0.91; 0.99[; ω_L2_ = 0.96, 95% CI ]0.85; 0.99[).

The team effectiveness second-order model had a good fit to the data (χ^2^(32) = 58.072, *p* = 0.003, *n* = 57; χ^2^/df = 1.815; *CFI* = 0.964; *TLI* = 0.950; *NFI* = 0.925; *SRMR* = 0.044; *RMSEA* = 0.120; *P*(rmsea ≤ 0.05) = 0.018, 90% CI ]0.068; 0.168[). The second-order reliability estimates were good (ω_L1_ = 0.92, 95% CI ]0.69; 0.97]; ω_partialL1_ = 0.95, 95% CI ]0.84; 0.99[; ω_L2_ = 0.97, 95% CI ]0.78; 0.98[).

##### Structural model

Because the used psychometric instruments (i.e., measurement model) presented good validity evidence based on the internal structure, a full structural model was tested for each of the related measures (i.e., team work engagement, team adaptive performance, and team effectiveness). The structural model that related team work engagement and shared mental models revealed an acceptable fit to the data (χ^2^(206) = 383.091, *p* < 0.001, *n* = 54; χ^2^/df = 1.86; *CFI* = 0.900; *TLI* = 0.888; *NFI* = 0.809; *SRMR* = 0.060; *RMSEA* = 0.126; *P*(rmsea ≤ 0.05) < 0.001, 90% CI ]0.106; 0.146[) with a strong and positive latent correlation (H4; *r*_team work engagement × shared mental model__s_ = 0.764; *p* = 0.099). The raw correlation between the arithmetic mean of the RSMMM’s items and the team work engagement’s items was strong and positive (*r* = 0.776; *p* < 0.001). The structural model that correlated shared mental models with team adaptive performance presented an acceptable fit to the data (χ^2^(185) = 329.269, *p* < 0.001, *n* = 54; χ^2^/df = 1.780; *CFI* = 0.916; *TLI* = 0.905; *NFI* = 0.829; *SRMR* = 0.038; *RMSEA* = 0.120; *P*(rmsea ≤ 0.05) < 0.001, 90% CI ]0.099; 0.141[) showing a positive strong latent correlation (H4; *r*_adaptive performance × shared mental model__s_ = 0.910; *p* = 0.053). The raw correlation was also strong and positive between the arithmetic mean of the RSMMM’s items and the team adaptive performance’s items (*r* = 0.888; *p* < 0.001). Finally, the model that correlated team effectiveness with shared mental models showed an acceptable fit to the data (χ^2^(226) = 423.832, *p* < 0.001, *n* = 54; χ^2^/df = 1.875; *CFI* = 0.895; *TLI* = 0.882; *NFI* = 0.801; *SRMR* = 0.050; *RMSEA* = 0.124; *P*(rmsea ≤ 0.05) < 0.001, 90% CI ]0.106; 0.142[) revealing positive and strong latent correlation (H4; *r*_effectiveness × shared mental model__s_ = 0.909; *p* = 0.068). The raw correlation between the arithmetic mean of the team effectiveness’ items and the RSMMM’s items was strong and positive (*r* = 0.857; *p* < 0.001).

Such correlation values suggest acceptable nomological evidence–particularly in terms of convergent validity evidence–in relation to the team work engagement scores. However, the correlation values between the shared mental models’ scores and the team adaptative performance and the team effectiveness seem too high (constructs overlap), providing poor convergent validity evidence.

Some of the presented models had mediocre *RMSEA* values. However, *RMSEA* point estimates depend on sample size, model degrees of freedom, and model misspecification (MacCallum et al., 1996; Chen et al., 2008). To assess the model’s fit to the data, other goodness-of-fit indices were presented in conjunction, namely, *SRMR*, which showed acceptable to good estimates. The *SRMR* goodness-of-fit index seems to be more robust than *RMSEA* across all conditions (Maydeu-Olivares et al., 2018).

## Discussion

There is a need to more fully examine the team dynamics present within professional sport referee teams (Aragão e Pina et al., 2018). In particular, given that shared mental models have been shown to play an important role in shaping team dynamics and performance in other context (Marks et al., 2000; Mathieu et al., 2000; Mascarenhas et al., 2005), there is a need to investigate the impact of referee teams shared mental models on team functioning and adaptability. However, the shared mental model literature suggests that one needs to adapt the measurement of such cognitive structures to the context within which such teams operate. As such, the primary aim of the current study was to develop a measure of shared mental models within the context of professional football and futsal referee teams.

The proposed new measure revealed good psychometric properties. Namely, the shared mental model measure developed in this article presented good validity evidence across the three different samples of football and/or futsal referees presented here. The RSMMM showed promising validity evidence both based on the internal structure and based on the relation with team work engagement (i.e., nomological evidence). Nomological evidence approaches the network of relations between the constructs. As so, the observed correlations between the latent variables (shared mental models and team work engagement) were aligned with the claims of the literature. Such findings suggest a useful unidimensional measure both for futsal and football referees.

The initial model (three first-order factors) revealed a lack of discriminant validity in terms of internal structure (Fornell and Larcker, 1981), indicating that the content explained by the three factors is similar. The dimensionality that emerged from the CD revealed that the referees on the sample perceive shared mental models as a unidimensional structure. Previous studies in which the RSMMM was based conceptualized it as a three-factor model (Santos et al., 2015a) or as a unidimensional one (Santos et al., 2015b). However, such solutions were not necessarily expected to be found in the referee context. Both the three-factor dimensionality of the SMMS (Santos et al., 2015a) and the unidimensional four-items version (Santos et al., 2015b) were proposed using a sample of teams from diverse contexts that participated on a virtual management challenge. Researchers have identified different dimensions of shared mental models including task, team, and strategy, as these are key aspects of the team work environment (e.g., Mohammed et al., 2010; Resick et al., 2010b). However, as Mohammed et al. (2000) state, “although the domain of a team model can vary (e.g., individual task work, team task work, team work), it should be viewed as reflecting how team members conceptualize a team-relevant phenomenon” (p. 125). Our study supports Mohammed et al. (2000) argument as our findings consistently suggest (over three different samples) that football and futsal referees have a general understanding of the relevant elements of team work and thereby do not distinguish between the different dimensions. Our findings are following previous studies that analyze the perception of shared mental models (Aubé et al., 2015, 2018; Santos et al., 2015b; Burtscher and Oostlander, 2019). Although conceptually, shared mental models may regard to different aspects of work, practitioners in a domain do not always seem to make this distinction, and results have supported a one-factorial solution (Aubé et al., 2015, 2018; Santos et al., 2015b; Burtscher and Oostlander, 2019). Mental models considerably derive from the occupational context in which they raise (Cannon-Bowers et al., 1993).

Additionally, the referees’ tasks are majorly concentrated during the referring of the futsal or football matches. Where everything is interconnected and must be deeply articulated between the team members, such contextual peculiarities might contribute to a perception of mental models as a singular whole. The suggested solution revealed a good fit for the single-factor model in three different samples of referees from two different sports (futsal and football), and as so, the H1 was supported. The RSMMM showed robustness in maintaining its dimensionality even when tested in a different sport other than football. Thus, such stability in the instrument’s structure allows for useful perspectives in terms of its implementation within other sports.

The second hypothesis was supported; thus, reliability evidence was good. The internal consistency estimates (i.e., α and ω) values were satisfactory for all the samples, based on the recommended values (Nunnally and Bernstein, 1994). Previous studies that used a similar measure also had good values of internal consistency estimates, namely, the unidimensional shared mental models proposed by Santos et al. (2015b), which had α = 0.92, and the PMU (which is another unidimensional measure) had α = 0.83 and ω = 0.83 (Burtscher and Oostlander, 2019). The TeamKMI internal consistency values of its five factors ranged from α_factor 3_ = 0.75 to α_factor 2_ = 0.89 (Johnson et al., 2007), whereas the SMMS had not its internal consistency values reported in its original study (Santos et al., 2015a). As such, the obtained results are aligned with previous studies using similar measures.

Measurement invariance among futsal and football referees was obtained. Such kind of psychometric property is essential to establish comparisons between mental model scores. Previous studies with referee samples using other instruments (e.g., Referee Self-Efficacy Scale) have not obtained full uniqueness measurement invariance among the sport referred; in fact, only partial factorial invariance was achieved (Myers et al., 2012). While studies using other instruments among football referees samples obtained different levels of measurement invariance, namely, metric invariance between referees and assistant referees (Brandão et al., 2014), and strong invariance (i.e., scalar invariance) among elite and non-elite referees (Johansen et al., 2018). An instrument’s mean scores should be compared only if scalar measurement invariance is granted (Marôco, 2014). Besides this fact, latent means comparisons should be implemented instead of raw means comparison, because the raw means do not account for measurement error. The established comparisons between shared mental models’ latent means were made only after the achievement of full uniqueness measurement invariance. Football referees perceived significantly higher shared mental models’ levels than futsal referees, which might be explained by the fact that in football the referee has a higher concentration of responsibilities in comparison with their assistants, whereas in futsal there is a higher sharing of those responsibilities between referees. As so, the perception of the shared mental models can be affected by the number of tasks with shared responsibility among the team members. The different levels of experience might also explain the differences between the shared mental models’ levels because the football referees had more years of experience than their futsal counterparts.

Regarding the validity evidence based on the relation to other variables, the RSMMM revealed acceptable nomological validity evidence in terms of convergent evidence with team work engagement. However, the convergent evidence was poor regarding team effectiveness and team adaptive performance. Thus, H4 was partially verified. The correlations among shared mental models with team adaptive performance; and shared mental models with team effectiveness were too high, suggesting some overlap of the constructs. The correlation among shared mental models with team work engagement was more adequate to the extent of convergence expected. Such empirical evidence allows confirming the expected direction of the associations with work engagement, reflecting partial support for the proposed theoretical nomological network (Lissitz and Samuelsen, 2007). This source of evidence was analyzed using study III’s sample (only composed by futsal referees), which might be a particularity of this small sample. Studies with small samples often report anomalously large effect sizes (Funder and Ozer, 2019), and as such, future replication studies might show that those effect sizes were overestimated with the used small sample (*n*_study III_ = 60) of futsal referees (Cumming, 2012).

This is the first instrument that explicitly measures shared mental models taking into consideration the specificities of football and futsal referee teams. All psychometric properties were indicative of good validity evidence, revealing a promising instrument for other contexts of referring (e.g., handball, basketball, rugby). The accumulated validity evidence seems to support the intended interpretation of the test scores for the RSMMM (American Educational Research Association, American Psychological Association, and National Council on Measurement in Education, 2014).

### Limitations and Directions for Future Research

While the current study provided some promising results about the RSMMM within the domain of football and futsal referees, there are some limitations in this study that must be acknowledged. For starters, this study was solely focused on referee teams within the sports of football and futsal. However, even though these are popular sports, it begs the question of how RSMMM would need to be altered to apply to other professional sport referee teams. Accordingly, it will be pertinent to see future research to examine the RSMMM in other sports and explore how this measure would need to be adjusted to be valuable and useful within other sports contexts.

In the present article, the data were analyzed at the individual level, not aggregated to the team level. Using the data aggregated to the team level could allow for a better understanding of the team’s global perceptions of themselves instead of the isolated individuals’ perceptions of the team. It is then possible to assess to which extent team members share mental models. For that propose, the level of agreement between team members would have to be considered for the subsequent analyses. It is worth mentioning that some of the referee teams are more stable in their constitution through the season than others (e.g., referees of lower categories tend to have more heterogeneity). The current cross-sectional study only provides a snapshot of the perceptions, which could vary if measured in a time frame (Levin, 2006).

The validity evidence based on the relations to other variables should be deeply investigated in terms of test criterion (e.g., higher team performance). As McNeese et al. (2015) urged, studying team cognition in sport must include a combination of both the shared knowledge and dynamical approaches. Future studies should investigate if shared mental models’ levels are associated with performance (e.g., match analysis report ratings or associations’ match/season ratings). Regarding the associations’ ratings, it might be also interesting to check which of the components of the assessment (there are usually three components: physical performance, performance of the written test regarding rules and the laws of the game, and performance of the match observations attributed by the referees observers) has higher association with shared mental models. The validity evidence based on the relations to other variables should also be investigated in terms of convergent (similar constructs) and discriminant evidence (measures purportedly of different constructs), preferably using different measures of other nature rather than perceptions (i.e., self-report measures). It is challenging to prove that representations exist beyond the boundaries of an individual organism and that such representations can be somehow shared with others. The use of technologies, as multiple eye tracker (Wildman et al., 2014) or hyperbrain networks (Filho et al., 2017), is encouraged. This kind of measures can surpass some of self-report measures limitations (Schwarz, 1999; Baumeister et al., 2007), particularly when it concerns measuring the perception of behaviors instead of behaviors (Lonati et al., 2018). With the robustness of such evidence, the RSMMM might give a step forward in its establishment as a measure of shared mental models among referees.

Study III’s sample size is small for structural equation modeling analysis, however, when looking to the number of futsal referees at the national level, it represents a considerable amount (30%) of the population of the Portuguese Football Federation (*N*_season 2018__–__2019_ = 177). Nevertheless, future studies should try to increase the number of referees both at the national and regional levels.

Additionally, given that in the collected samples were only a few football assistant referees (*n* = 25), the measurement invariance was not tested among them. Accordingly, given that assistant referees play an essential role within the football referee team, future studies should account for this and explore what impact having assistant referees more represented in future research samples can alter. Finally, within the current study and the underlying data that were used here, measurement invariance across time could not be examined. As a result, no statements regarding the trends that may exist across time can be made. In response, future research should examine this fact and collect the type of data necessary to be able to assess longitudinal measurement invariance. The assessment of validity evidence is an ongoing and never-ending process (Slaney, 2017); thus, the next steps should seem like a natural on the evolution of the RSMMM as an established measure to approach shared mental models within referees.

## Data Availability Statement

The raw data supporting the conclusions of this article will be made available by the authors, without undue reservation.

## Ethics Statement

Ethical review and approval was not required for the study on human participants in accordance with the local legislation and institutional requirements. The patients/participants provided their written informed consent to participate in this study.

## Author Contributions

JorS, JP, and AP contributed to conception and design of the study. JorS, JP and JoãS organized the database. JorS and JM performed the statistical analysis. JorS wrote the first draft of the manuscript. JP, JoãS, and CS wrote sections of the manuscript. All authors contributed to manuscript revision, read and approved the submitted version.

## Conflict of Interest

The authors declare that the research was conducted in the absence of any commercial or financial relationships that could be construed as a potential conflict of interest.
